# MICRO-FRAGMENTED ADIPOSE TISSUE IN THE KNEE OSTEOARTHRITIS UNDER LOCAL ANESTHESIA

**DOI:** 10.1590/1413-785220253301e287060

**Published:** 2025-02-03

**Authors:** Bruno Butturi Varone, Henrique Fuller, Daniel Perini, Daniel Peixoto Leal, Riccardo Gomes Gobbi, Marco Kawamura Demange

**Affiliations:** 1 Universidade de Sao Paulo, Faculdade de Medicina, Hospital das Clinicas HC-FMUSP, Sao Paulo, SP, Brazil

**Keywords:** Osteoarthritis, Knee, Subcutaneous Fat, Knee Joint, Osteoartrite do Joelho, Gordura Subcutânea, Articulação do Joelho

## Abstract

**Objective:**

To assess the feasibility of the entire micro fragmented adipose tissue knee injection procedure under local anesthesia. From the subcutaneous harvesting and microfragmentation process to the intrarticular knee injection.

**Methods:**

A patient with bilateral knee osteoarthritis underwent adipose tissue harvesting and bilateral intra-articular micro fragmented adipose tissue knee injection under local anesthesia. Patient-related outcomes were collected before the procedure, 12 months, and 24 months follow-up. Womac, Koos, and VAS were recorded.

**Results:**

The visual analog scale, KOOS questionnaire, and WOMAC score all improved in the 12- and 24-month follow-ups.

**Conclusion:**

Adipose tissue harvesting and micro fragmented adipose tissue knee injection are procedures that can be performed under local anesthesia and have good patient report outcomes. ***Level of Evidence IV; Therapeutic Study.***

## Resumo

**Objetivo:**

Avaliar a possibilidade da realização do procedimento de injeção intra-articular de tecido adiposo microfragmentado sob anestesia local. Desde a coleta subcutânea até o processo de microfragmentação e injeção intra articular.

**Métodos:**

Um paciente com gonartrose bilateral que foi submetido a retida de tecido adiposo e injeção intra articular nos joelhos de tecido adiposo microfragmentado sob anestesia local. Desfechos relacionados ao paciente foram coletados antes do procedimento, no seguimento após 12 e 24 meses. Womac, Koos, and VAS foram avaliados.

**Resultados:**

Escala visual de dor, questionário KOOS e WOMAC todos mostraram melhora nas visitas de seguimento aos 12 e aos 24 meses.

**Conclusão:**

Coleta de tecido adiposo e injeção de tecido adiposo microfragmentado no joelho é um procedimento que pode ser realizado sob anestesia local com bons resultados reportados pelo paciente. **Nível de Evidência IV; Estudo Terapêutico.**

## INTRODUCTION

Knee osteoarthritis (KOA) is one of the most prevalent joint diseases,^1^ affecting more than 13% of men and 10% of women over 60 years of age.^2^ The prevalence is expected to increase with the advancing age of the population and the prevalence of obesity.

The treatment for KOAs ranges from conservative treatment with physiotherapy and muscle strengthening to a surgical approach with total knee arthroplasty. Recently, biological and regenerative therapies have begun to provide new perspectives within orthopedics. These therapies can expand the non-surgical or minimally invasive options available as a treatment for patients with early OA.^3^ The orthobiologicals are found naturally in the human body. The most studied orthobiologicals currently are: platelet-rich plasma (PRP), hyaluronic acid(HA), microfragmented adipose tissue (mFAT) and bone marrow aspirate concentrate (BMAC).

The use of mFAT in the context of knee osteoarthritis has been studied due to the large availability of tissue and easily accessible tissue for harvesting. Encouraging results in mild to moderate cases in a 3-year follow-up,^4^ and even in severe KOA a the short-term significant improvement (1-year follow-up) in the KOOS and WOMAC scales.^5^ Performing this procedure on an outpatient basis under local anesthesia has been increasingly encouraged, once more patients would benefit from the increased availability of this therapy. Until the present moment, the vast majority of mFAT collection procedures have been performed under general anesthesia without major complications.^6^

## OBJECTIVES

Several studies have shown the beneficial effects of intrarticular injection of mFAT for degenerative conditions, especially for knee osteoarthritis.

Harvesting adipose tissue from the low abdominal area can appear challenging for orthopedics surgeons. Studies show that a small volume of adipose tissue is necessary to prepare micro fragmented adipose tissue (mFAT) for an articular injection.

Therefore, the objective of this study is to report a 24-month follow-up case of mFAT infiltration performed just under local anesthesia.

## METHODS

This protocol case was done preliminarily to access the feasibility of harvesting adipose tissue, microfragment the subcutaneous tissue with a one-stage device Lipogems® and injected the mFAT intra-articularly in both knee all in an ambulatory setting. The study was approved in the ethics committee number 5.259.237. The patient signed an informed term of consent.

### Patient demographics

Our pilot patient is a 56 year old female who states that has bilateral pain for over 6 years. She failed initial non-operative treatment with physical therapy, analgesics and anti-inflammatory medications. She is otherwise healthy, her BMI is 26,5kg/m^2^.

According to Kellgreen Lawrence classification, right knee was considered grade 3 and left knee was considered grade 4.

### Patient set up

The patient underwent a bilateral knee injection. Adipose tissue was collected under sterile conditions in a surgical center under local anesthesia, without sedation. The attire included a hat, gloves, private clothing, and face masks. The participant was positioned in the supine position on a surgical table. In all patients, an intravenous access was obtained for the administration of cefazolin 2g as antibiotic prophylaxis, sodium dipyrone 1g and dimenhydrate 50mg.

### Local anesthesia

**Figure 1:**
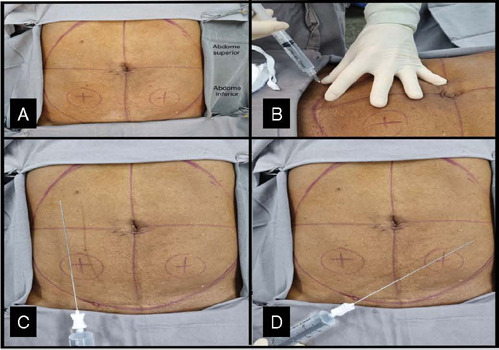
Patient positioning, portal placement.

Figure 1. Patient positioning, portal placement.

Adipose tissue (AT) harvest site was performed in the lower abdomen. The portals were marked above the inguinal line, on each side of the abdome.

The skin anesthesia of 1ml of 2% lidocaine was applied in the portal location. After the anesthetic latency time, a small incision of approximately 4 mm was made with an 11-blade scalpel on each side of the abdomen. For harvesting the adipose tissue, a two staged intumescent technique was performed.

The technique for anesthetic infiltration consists of inserting the cannula through the portal made in the skin to disperse the solution throughout the area of capture of subcutaneous adipose tissue. First, subcutaneous adipose tissue was infiltrated through a 19G cannula provided by the Lipogems® kit.

The injected solution was composed of 20 ml of 2% lidocaine, 20 ml of 0.5% bupivacaine, 1 ml of 1mg/ml adrenaline and 250 ml of 0.9% saline. Totaling a volume of 291ml. We used 120ml of this solution in each hemi-abdome, the remaining 51 ml was reserved to be used in case we needed more anesthesia during the harvesting. Since the procedure was performed with no sedation, some details are important: A 20ml syringe was used to diffuse the solution in a low-flow and low-pressure way. The subcutaneous tissue intumescence was carried out slowly to avoid discomfort to the patient.

The capture region must be homogeneously infused so that tissue harvesting does not cause discomfort.

Based on the latency of lidocaine, we standardize a 5-minute wait before proceeding with the adipose tissue harvesting. After the waiting period, the adipose tissue harvesting was performed with the 13G cannula provided in the Lipogems® kit.

### Adipose tissue harvesting

A vaclock syringe was connected to the cannula, this syringe is specially designed to keep adequate pressure in the system for an optimal fat tissue harvest.

Adipose tissue was harvested in a homogeneous way, avoiding repetitive harvesting next to the portal, which can lead to cosmetic problems. Pinching the abdomen to evaluate the amount of remaining subcutaneous tissue in each area is a reliable way to avoid any cosmetic changes.

We planned to harvest 120ml of adipose tissue which would lead to approximately 20ml of mFAT. Our goal was to inject 10ml of mFAT on each knee.

After the procedure, skin portals were closed with Nylon 5.0 sutures, and Adipose tissue was processed using Lipogems®, a single-use and disposable kit.

**Figure 2:**
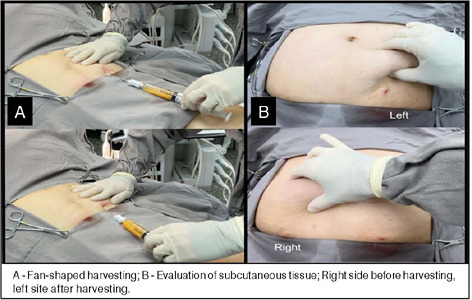
Adipose tissue harvesting

Figure 2. Adipose tissue harvesting.

A - Fan-shaped harvesting; B - Evaluation of subcutaneous tissue; Right side before harvesting, left site after harvesting.

### Adipose tissue processing

Adipose tissue was processed using Lipogems®, a single-use and disposable kit that through mild mechanical forces, washing, and reduction filters eliminates oil from ruptured adipocytes and red blood cells present in the adipose tissue aspirate.

**Figure 3:**
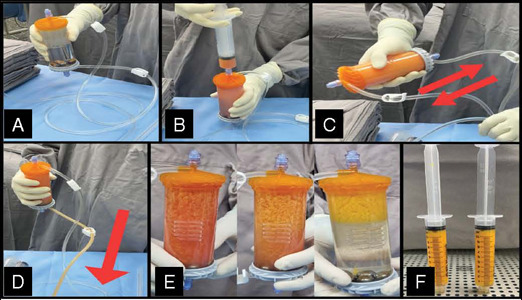
Adipose tissue micro-fragmentation procedure.

For this patient, 120 ml subcutaneous tissue was inserted into the system, which was prefilled with saline. After that, mechanical dissociation was obtained by gently shaking the system. Stainless steel marbles inside provide additional mechanical fragmentation. Oil residues and blood components are washed out by gravity counter-flow of saline solution, this procedure is repeated until the solution in the divide appears clear and the lipoaspirate is yellow. Microfragmented adipose tissue migrates to the top of the device. Finally, the device is turned upside down by 180 degrees, with the fat tissue product now facing a narrower size reduction filter. MFAT is then removed from the device and reserved in 10-ml syringes. Figure 3. MFAT processing

A - The system is filled with saline; B - harvested adipose tissue is inserted; C - micro-fragmentation due to gentle mechanical forces; D - fat is washed and rinsed; E - changes in the aspect after the washing procedure; F - final product.

### Ultrasound-guided intra-articular infiltration

With the patient in the supine position, the knees were prepared with 2% degerming chlorhexidine gluconate, then cleaned with an alcoholic chlorhexidine solution, in addition to placing sterile drapes. The area was anesthetized with a 1ml anesthetic button and deep tissues were anesthetized with an additional 1ml of 2% lidocaine. A 16G Jelco needle was inserted into the suprapatellar bursa under the guidance of a Logiq E GE Healthcare® ultrasound device and a 12MHz linear probe to ensure that the infiltration of the product was articular. If there was a joint effusion, the excess fluid was drained. The microfragmented fat tissue was then infiltrated. The orthopedic surgeon who performed the joint infiltration has experience in the area, and the use of ultrasound during infiltration increases the precision and degree of certainty that the product was delivered to the joint cavity.

**Figure 4:**
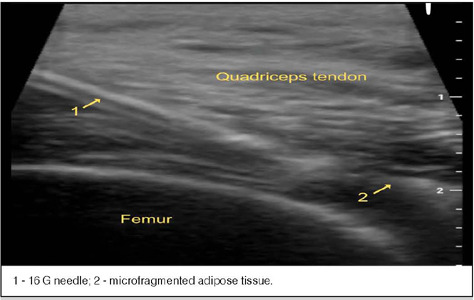
US guided injection.

Figure 4. US guided injection

1 - 16 G needle; 2 - microfragmented adipose tissue.

### Post-operative care

The patient was admitted and discharged on the same day. The participant was instructed to remove all dressings 24 hours after the procedure. Stitches were removed on a seven-day follow-up. Patients was instructed to avoid physical activities or more intense efforts for one week. The home prescription consisted of dipyrone sodium in cases of mild pain and tramadol in cases of more intense pain. Hirudoid® (mucopolysaccharide polysulfate) was recommended in its topical gel form to minimize bruising. Furthermore, cryotherapy was indicated to control pain, edema, and bruising.

### Outcomes

Patient-reported outcomes (WOMAC, VAS, and KOOS) were accessed at the baseline and in the 12 and 24-month follow-ups. The patient was evaluated within seven days of the procedure to evaluate adverse effects due to adipose tissue harvesting and the bilateral knee injection. The patient was instructed to report any discomfort or pain to the medical team.

## RESULTS

During all those 24 months of the follow-up period, there were no complications regarding the harvest site and injected knees. It was observed at the 7-day follow-up, as expected, mild abdominal ecchymosis and knee discomfort that resolved within 15 days from the surgery. No important cosmetic changes were identified during the 24 months.

All three parameters showed an improvement on both the first and second follow-up appointments (Table 1). The WOMAC score^7^ questionnaires before the procedure was 83, on the 12-month follow up it was 24, and on the 24 month follow up 30. Changes in values greater than 10 are above minimal clinically important difference (MCID)^8^ and changes greater than 25 are above substantial clinical benefit.^9^ The baseline VAS score was 8, after 12 months VAS was 1, and on the 24-month follow up 3 points. KOOS questionnaire was 38% at the baseline, 54% at 12 months, and 50% at 24 months follow-up, both greater than MCID when compared to baseline score.^10^

**Table 1:** Results at baseline, 12 month follow up and 24 month follow up.

	Womac score (0-96)	VAS	KOOS
Preop	83	8	38%
12 months	24	1	54%
24 months	30	3	50%

## CONCLUSION

Most studies had evaluated the feasibility of harvesting adipose tissue for obtaining mFAT associated with other procedures such as osteotomy,^4^ knee arthroscopy,^6^ or meniscus tears.^11^ Therefore, in these cases, the harvesting is usually performed with the patient under sedation. Knee osteoarthritis is an extremely prevalent disease, and mFAT has recently shown its beneficial effect in this disease.^5^,^12^,^13^ Evaluating the safety and feasibility of this procedure in an ambulatory setting is essential to make mFAT treatment available to more patients. In this case report, we showed that, with adequate technique, it is possible to perform this procedure under local anesthesia in patients with knee osteoarthritis. We also report important symptomatic and quality of life improvements in the patient, shown in her patient-reported outcomes improvements. Those results encourage further studies using this setting in controlled protocols with a larger number of patients.
